# Analysis of conventional and helical soil nails using finite element method and limit equilibrium method

**DOI:** 10.1016/j.heliyon.2022.e11617

**Published:** 2022-11-17

**Authors:** Archita Goyal, Amit Kumar Shrivastava

**Affiliations:** Department of Civil Engineering, Delhi Technological University, India

**Keywords:** Soil nailing, Conventional soil nail, Helical soil nail, Slope stability, Finite element method

## Abstract

The infrastructure of roads, bridges, tunnels, subways, etc. is grooming day by day. In the recent era, soil nailing is the developing method for slope stability, road widening projects, and constructing deep foundations. This paper suggests a new and improved helical soil nail (HN) that offers an alternative to conventional soil nail (CN). Till now pullout studies on different model nails in c-Φ soil are still rare. In addition, the stability analysis for conventional soil nails was only studied using FEM analysis, whereas for helical soil nails the estimation of stability of slope is still mysterious. So, the present study includes the behavior of conventional and helical soil nails based on both laboratory and analytical methods in c-Φ soil. The present study also incorporated the use of theoretical equations and finite element analysis (using PLAXIS 2D) for comparing the stability of different profiles of soil nails (e.g., CN and HN) in c-Φ soil, which was unique to this study. By using the finite element method (FEM) and the limit equilibrium method (LEM), a factor of safety was also estimated for both types of nails (CN and HN). According to the experimental, analytical, and numerical findings, it is clear that helical soil nails (HN) exhibit less deformation and more factor of safety than conventional soil (CN) nails under similar loading and soil conditions.

## 1 Introduction

The soil nailing procedure is extensively been employed to support the unstable soil mass for the last two decades [1], [2], [3], [4]. The strength of unstable cuts can be increased by installing soil nail elements to the soil mass. The soil nail element offers passive resistance due to soil-nail interaction, which ultimately increases the soil nail pullout capacity [5], [6], [7], [8], [9], [10], [11], [12], [13], [14]. The pullout is taken into account as the most essential parameter for the design of soil-nail walls [16], [17], [18], [19], [20]. Also, soil nail elements are easy to apply in congested places, economical, and have good performance under dynamic conditions [5], [6], [7], [8]. When the helical soil nail is considered, a group of helices was attached along the shaft length as part of the advancement in the process, resulting in a better version of the conventional soil nail (CN). Helical soil nails (HN) are a newer type of soil nail that employs helical bearing to provide higher pullout strength while causing less soil disturbance [20], [21], [22], [23], [24]. A helical soil nail is a passive element that depends on the drive of the unstable soil mass to mobilize the shear stress along the nail length. This technique is a cost-effective and realistic solution for earth retention work [5], [25]. Various researchers have investigated the laboratory and numerical study on both conventional and helical soil nails in cohesionless soil only. Rawat and Gupta [8] compared the interface friction of CN and HN in sandy soil. Further, applied numerical methods were for the analysis of different parameters of both CN and HN in frictional soil. An experimental study was conducted on model solid and hollow shaft helical soil nails with varying geometric parameters of the specimen recommends helical soil nails over conventional soil nails [11]. In the past decade, different laboratory and analytical studies were conducted on different model soil nails in only cohesionless soil (Φ-soil) [18], [19], [20]. In actual practice, hardly the case there is only cohesionless soil in any soil slope, embankment, or landmass. However, pullout studies on different model nails in c-Φ soil are still rare to the best of the author's knowledge. In addition, till date, the stability analysis for conventional soil nails was only studied using FEM analysis [20], [21], [22], [23], whereas for helical soil nails the estimation of stability of slope is still mysterious. As a result of the gaps, the current study's goals are to investigate the load-displacement behavior of different nail specimens in c-Φ soil. The current study explored the behavior of soil nails based on both laboratory and analytical methods in c-Φ soil. The present study also incorporated the use of theoretical equations and finite element analysis (using PLAXIS 2D) for comparing the stability of different profiles of soil nails (e.g., CN and HN) in c-Φ soil, which was unique to this study.

## 2 Physical model setup

### 2.1 Material and sample preparation

**Figure 1:**
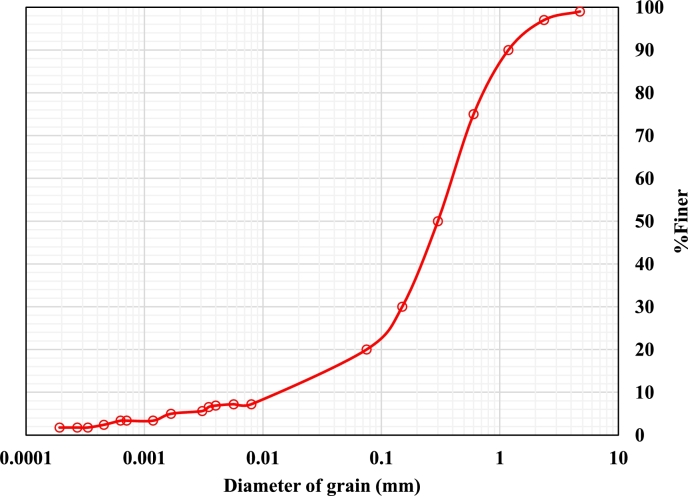
Grain size distributions.

The locally available soil was taken from the area near Ajay Kumar Garg Engineering College, Ghaziabad, India. The sieve and hydrometer analysis were carried out on the soil sample as per IS: 2720, Part-4 [15]. C_u_ and C_c_ were found to be 8.18 and 1.78, respectively, in the test findings. The gradation curve indicates that the soil is well-graded sand (C>u6) as shown in Fig. 1 and fines in the soil are more than 12% therefore soil attains dual naming, classified as well-graded sand with clay (SW-SC).

**Figure 2:**
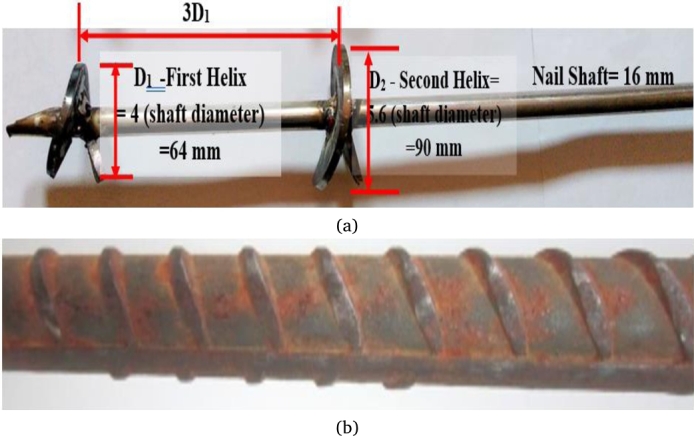
Helical Soil Nail.

Table 1 lists the geotechnical properties of the soil material. Mild steel was used to fabricate the nail specimens shown in Fig. 2(a) and Fig. 2(b). The model nail specimen's diameters were selected after scaling down the original diameter using Eqn. (1) [8], [9], [10], [11].

(1) $$d_{m}=\frac{d_{p}}{k}$$

**Table 1:** Geotechnical properties of soil sample.

Property	Value
Specific Gravity, Gs	2.65
D10 (mm)	0.055
D50 (mm)	0.30
D30 (mm)	0.21
D60 (mm)	0.45
Coefficient of curvature, Cc	1.78
Coefficient of uniformity, Cu	8.18
Friction angle, Φ (°)	31^0^
Cohesion, c (kN/m^2^)	22
Relative density, (RD)	65%
ϒd(min) (kN/m^3^)	13.13
ϒd(max) (kN/m^3^)	16.87

Where d_m_ is the model nail diameter

d_p_ is the prototype nail diameter,

k is the scale factor

Both conventional and helical soil nails have a 16 mm diameter shaft, whereas the helical diameter is 4 to 6 times the shaft diameter [9], [10], [11], [12], [13], [14] shown in Fig. 2(a) and Fig. 2(b). In this study, the effective length of nails was considered 0.7 m, whereas the length of the nail specimen was 1 m. “No scale impact has been observed if the ratio of minimum shaft diameter (d_s_) to mean grain size of soil (D_50_) and effective radius of a helix (E_r_) to mean grain size of soil (D_50_) are more than 30 and 58, respectively” [9], [10], [11], [12], [13], [14]. In the present study, d_s_/D_50_, and E_r_/D_50_ are equal to 53.33 and 123.3, respectively. The scale factor adopted in the present study was 5.55 to satisfy the no-scale effect condition. Hence, the laboratory results will be unaffected by the scale effect. The tank size adopted in the present study was 2000 mm in length, 1100 mm wide, and 1100 mm high respectively. To eliminate the boundary effect of the model tank, the tank dimension was chosen in such a way that the lowest dimension of the tank is selected as ten times greater than the maximum helical diameter [9], [10], [11], [12], [13], [14]. The soil sample was compacted in the test tank in ten layers up to 1000 mm in height with each layer of 100 mm thickness. Further, the sample was placed under a steel plate of 8 mm thickness under overburden pressure of 10 kPa, 20 kPa, and 40 kPa respectively to know the behavior of nails under different pressure. The important parameter pullout resistance significantly depends upon the overburden pressure. Both the soil nails were installed under similar conditions i.e., the presence of similar overburden pressure. Under very high-pressure soil undergo an arching effect resulting decrease in pullout resistance [22], [23], [24] thus large soil slope is sub-divided into small segments during the execution of soil nail in the actual field also to reduce the high overburden pressure. Based on this recommendation, a different range of surcharge pressure was adopted by a few researchers as given in Table 2. Therefore, in the present study low confining pressure range of 10 kPa, 20 kPa, and 40 kPa was employed using a hydraulic jack.

**Table 2:** Different ranges of overburden pressure used by researchers during experiment.

S. No.	Overburden pressure	Reference
1	5.6 to 22.7 kPa	Milligan GWE, Tei K (1998)
2	0-150 kPa	Pradhan et al. (2006)
3	5 to 50 kPa	Sharma et al. (2020)

### 2.2 Nail installation and pullout

**Figure 3:**
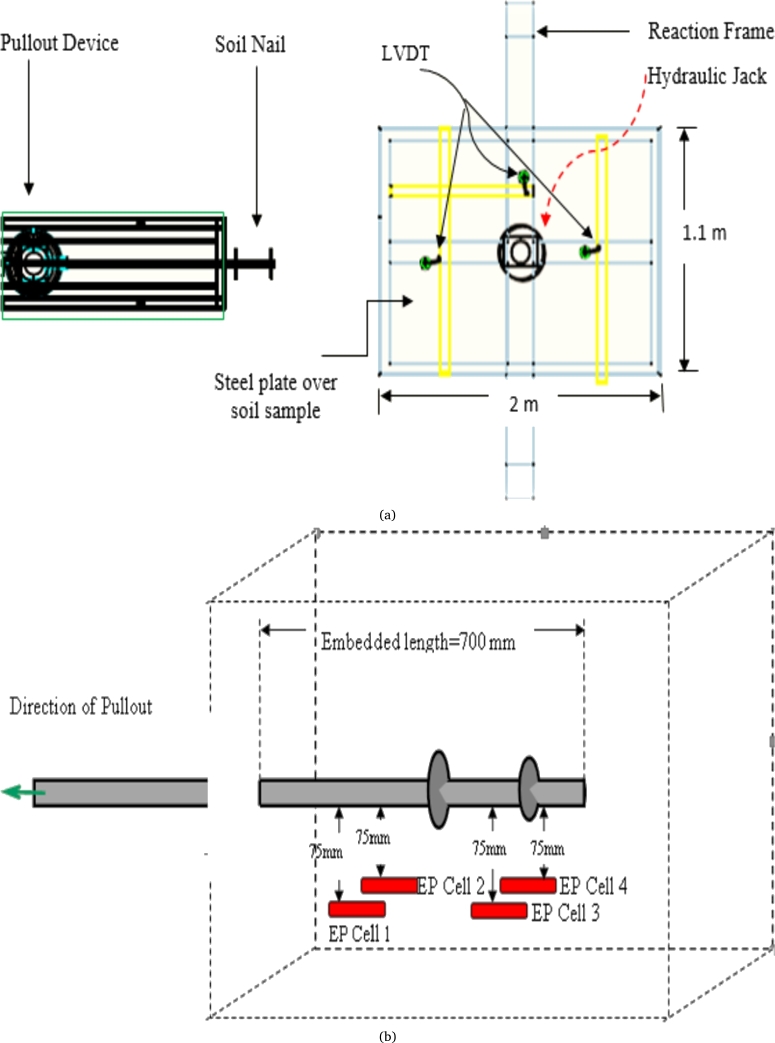
(a). Schematic diagram of laboratory Pullout device. (b). A pictorial illustration of the location of the earth pressure cell.

The uniqueness of this work is that the installation purpose of CN and HN, methods followed are similar to the method used for the installation of these nails in the field. For conventional soil, the trench was drilled near the center at a height of 0.5 m from the base using a drilling machine (0.5 horsepower). As per Federal Highway Administration, Washington, 2015, it is highly suitable to install a soil nail from 0° to 20°, while 15° is considered the most optimum angle for the installation of a soil nail. However, due to ease of installation purpose or site condition, one can adopt any angle between 0° to 20°. The conventional nail is then inserted up to 700 mm depth and then the grouting operation was executed under gravity flow (without pressure). Thus, pressure remains unchanged after the grouting operation. The water-cement ratio used in the grouting operation of CN was 0.55. For the installation of HN, the nail was installed at a revolution rate of 10 RPM with a 10 mm/min forward controlled displacement rate. The pullout of both CN & HN was measured by the displacement-controlled machine having a maximum pullout capacity of 50 kN (Fig. 3a) [9], [10]. The pullout displacement rate for both types of nails adopted in the study was 10 mm/minute [1]. The bond failure value is calculated when the increase in resisting force per 1 mm displacement is less than 1%, or when the displacement exceeds 30 mm [18], [19].

For calibrating earth pressure cells and load cells, a perspex cylinder sealed with water and a pressure controller was used (in the industry). The data logger was utilized to record the earth pressure/load cell's response to each increment of water pressure ranging from 0 kPa to 100 kPa in the cylinder. Installation operation of soil nails causes disturbances to the soil mass; thus, the installation stage is one of the important phases of execution. The installation process was monitored by using earth pressure (EP) cells around the soil nail as shown in Fig. 3(b). The capacity and sensitivity of EP cells are 3 MPa and 0.01 MPa respectively. For the installation of conventional soil nails, a drill chuck was used to drill the soil nail cell. During drilling, significant changes in internal stresses were recorded.

## 3 Mathematical model of load-displacement behavior

The mathematical modeling of soil nail load-displacement behavior aids in verifying or determining the bond strength of the soil nail wall system. The hyperbolic model of soil nails subjected to some external force is based on the equilibrium forces. The curve is divided into two sections, i.e., pre-peak and post-peak behavior. The mathematical models for soil nails subjected to external loading are adopted by Sharma et al. [11]. The expression for the pre-peak and post-peak stage of both CN and HN are given as follows:

The expression for the pre-peak stage of pullout force (F) depends upon the surface area of the soil nail and interface friction of soil and nail respectively. The equation of load displacement is designed from the equilibrium equation of different forces. Based on the equilibrium equation the derived equation for pullout force is given in Eqn. (2).

(2) $$F\left(x\right)=\frac{\pi }{4}D_{cs\;or\;h}^{2}E\epsilon (x)$$

where F(x) is externally applied force on soil nail;

$D_{cs\;or\;h}$ is shaft diameter for CN and helical diameter for HN;

E is known as the modulus of elasticity of soil-nail;

ϵ(x) is the axial strain at point x,

A differential equation of second order is derived which expresses the relationship between pullout force and the displacement in Eqn. (3)

(3) $$\frac{\partial^{2}F(x)}{\partial x^{2}}=\frac{4KF(x)}{\pi^{2}ED_{csorh}^{3}\tau_{ult}^{2}}{\left[\frac{dF(x)}{d(x)}+\pi D_{h}\tau_{ult}\right]}^{2}$$

In the present study, the theoretical details are adopted from Sharma P [25], which is solved by using Wolfram Mathematica 7.0 by applying suitable boundary conditions as expressed in Eqn. (4) and (5).

(4) $$F(x=0)=F_{0}$$

(5) F(x=L)=0

$F_{0}$ is Null force at 0% displacement;

L is the length of the nail;

In the post-peak stage for CN and HN, the residual factor was calculated using Eqn. (6) [25].

(6) $$f=\frac{\tau_{p}-\tau }{\tau_{p}-\tau_{r}}$$

$\tau_{p}$ is the peak value of shear stress;

$\tau_{r}$ is the residual value of shear stress;

*τ* is primary shear stress.

Whereas the change in residual factor with displacement is given in Eqn. (7) for the post-peak stage [11], [25].

(7) f=0.97ln[u(x)]−3.12

The post-peak value was calculated using Eqn. (10) and (11) under varying surcharge pressure.

## 4 Result and discussion

### 4.1 Pullout behavior

**Figure 4:**
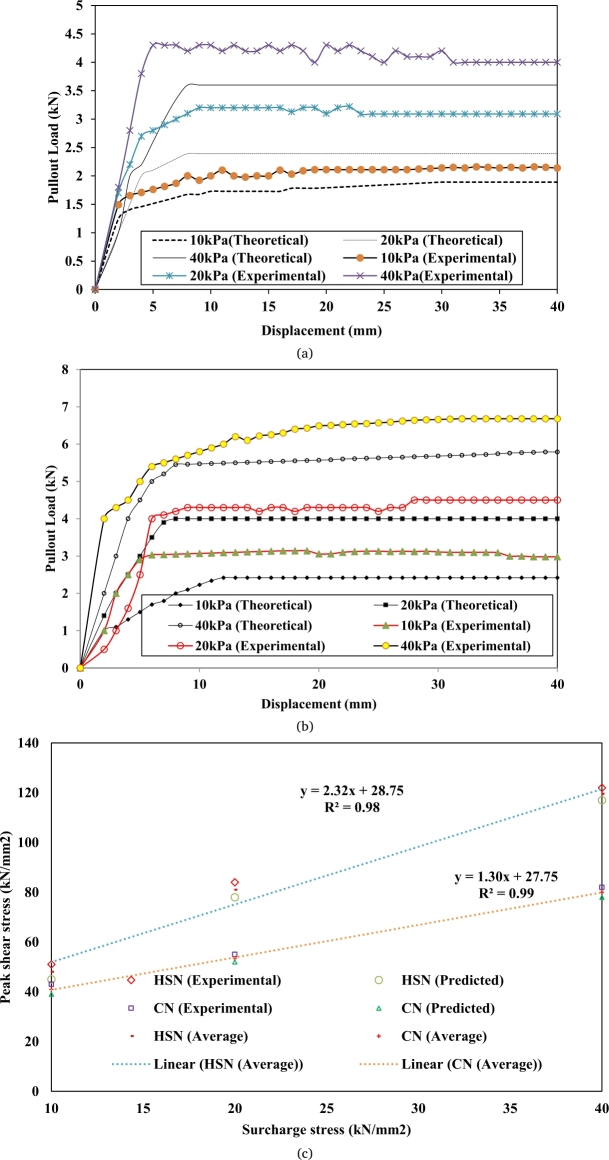
(a). Experimental and simulated (theoretical) load-displacement behavior of conventional soil nail in c-Φ soil. (b). Experimental and simulated (theoretical) load-displacement behavior of helical soil nail in c-Φ soil. (c). Experimental and Predicted shear stress.

From the laboratory test performed over the c-Φ soil using the displacement-controlled machine, evident from Fig. 4(a) and Fig. 4(b) as the external force is directed to the nail head, the nail starts offering significant resistance with displacement in its initial stage. With the further application of force, it achieves its peak and then the force starts decreasing with further displacement for both CN and HN. As evident from Fig. 4(a) and Fig. 4(b), as overburden pressure increases over the soil nail it starts offering extra pullout resistance.

The pullout resistance provided by both CN and HN is exactly proportional to overburden pressure, according to the results of the test. The reason for this is that when the surcharge value rises, the soil-nail interaction and adhesion value rise, increasing the overall resistance force of the soil-nail. Because of the increased shear stress surrounding the nail, the soil nail's pullout capacity is increased. So, the helical soil nail offers more resistance than conventional soil nails under varying surcharge pressure. An experimental study shows that helical soil nails have a 42% greater pullout capability than conventional soil nails under varying surcharge pressures. Based on the mathematical model of the load-displacement behavior of a soil nail element the predicted results follow a similar trend, however, the predicted result shows a slightly lesser value than the experimental value. The reason may that in mathematical modeling the result is based on soil nail stiffness value or interface friction value. The mathematical model is not able to incorporate the installation effect and lateral pressure in the model. The other possible reason for the difference in measured and calculated pull-out forces may be due to the impact of different design parameters. The pull-out of soil nails depends on nail shaft type, helix parameter, the surface of the shaft, shear strength parameter of soil, friction angle, etc. Pull-out testing of soil nails may also cause a soil dilatancy effect after slight displacement of the soil nail [16], [17], [18], [19]. As a result, the model predicts a lower number, but it follows the same trend as the experimental value. The experimental value is in good agreement with the predicted values. The predicted results are on the conservative side; hence the mathematical model can predict the approximate value of the pullout load results in the absence of laboratory testing. The pullout strength can also be studied in expressions of shear stress i.e. $\left[\text{Shear stress}=\frac{\text{Pullout force}}{\text{surface area of the nail}}\right]$, thus shear stress on soil nails is proportional to the pullout force of soil nails. The peak shear stress under different surcharge pressure was plotted against varying overburden pressure. Both experimental and theoretical values follow a similar pattern and increase with the surcharge pressure. The pullout resistance or pullout shear stress follows a linear relationship with surcharge pressure, which shows that pullout force follows the Mohr-Coulomb trend (Fig. 4c).

Based on the experimental and theoretical values an average value of pullout shear stress was drawn and generated a relationship to predict pullout force and pullout shear stress as well under different overburden for both CN and HN respectively as given in Eqn. (8) and Eqn. (9).

For conventional soil nails:

(8) Peak Pullout capacity or shear stress=1.3q+28

For helical soil nails:

(9) Peak Pullout capacity or shear stress=2.32q+29

where q is overburden pressure.

**Figure 5:**
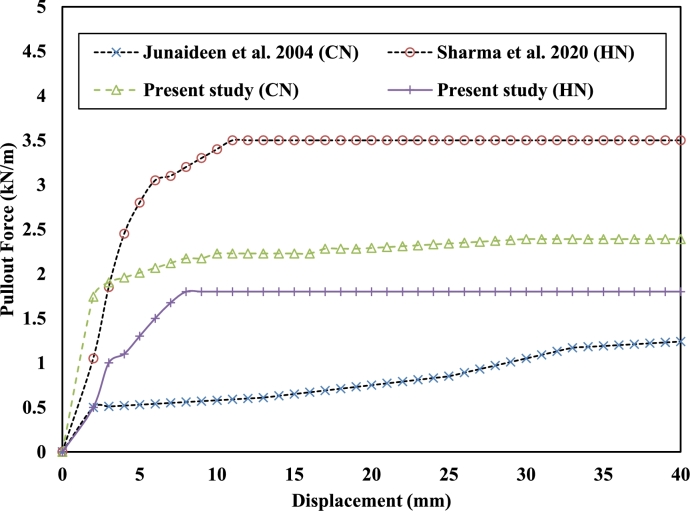
Comparison of load-displacement behavior of present study with literature.

Based on laboratory tests and predicted results Eqn. (8) and Eqn. (9) were generated for the calculation of peak pullout capacity or peak shear stress with regression values of 0.99 and 0.98 for CN and HN respectively. To validate the present study, the experimental data from previously published literature have been presented in Fig. 5. The laboratory results of this study have been compared with previously published experimental results for different soil nails and soil conditions. The load-displacement curve shows for both the present study and previously published results follow similar trends [5], [6], [7], [8], [9], [10], [11], [12].

### 4.2 Results for internal soil stresses

**Figure 6:**
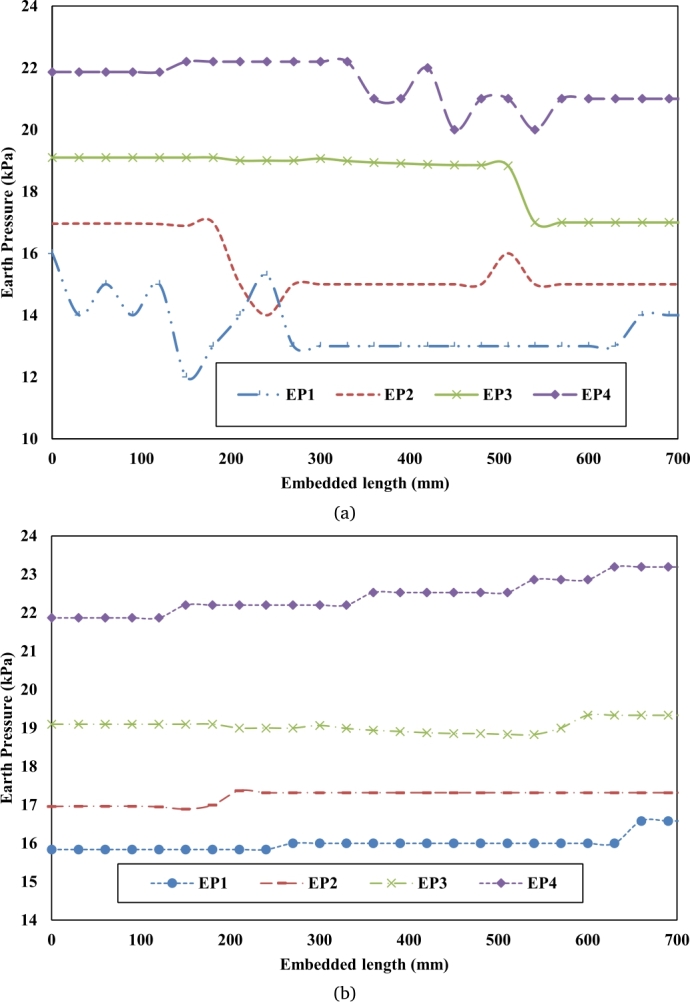
(a). Earth pressure during installation of conventional soil nail. (b). Earth pressure during installation of the helical soil nail.

As evident from Fig. 6(a) with an increase in installation depth or embedded length significant change in earth pressure was recorded by each set of cells. This change in earth pressure represents a significant change in the volume of soil mass during the installation of CN. Evident that during the installation of CN significant disturbances are generated. Further, with an increase in installation depth, the internal stresses decrease continuously. The internal stresses remain almost unchanged even after the grouting of CN. During the installation process of the helical soil nail, a continual increase in earth pressure was seen in Fig. 6(b)., indicating that the advancing movement of the HN caused a significant increase in confining pressure resulting in a rise in the density of soil mass around the periphery of the nail. Ultimately the soil mass will provide more pullout force around the soil nail. The variance in earth pressure is caused by the non-homogeneous nature of the soil, which contains particles ranging from coarse sand to clay, each with a distinct unit weight, causing variations in earth pressure [10], [11], [12], [13].

### 4.3 Factor of safety of CN and HN

In a soil-nailing system, the factor of safety (FOS) depends on the geometry, shear strength parameters of the soil, and nail material. According to FHWA [1], the Limit Equilibrium Method (LEM) will be a safe way to design soil slopes using Eqn. (10).

(10) $$FOS_{N}=\frac{\left(F_{r}\cos\left(\Psi -i\right)+\left\{\left(w+q\right)\cos\Psi +F_{r}\sin\left(\Psi -i\right)\right\}\tan\Phi \right)}{\left(w+q\right)\sin\Psi }$$

where,

$F_{r}$ is equivalent nail force = $0.75K_{a}\gamma_{s}zS_{h}S_{v}$;

*γ* is the unit weight of soil;

S_h_ is the horizontal spacing

S_v_ is vertical spacing

**Figure 7:**
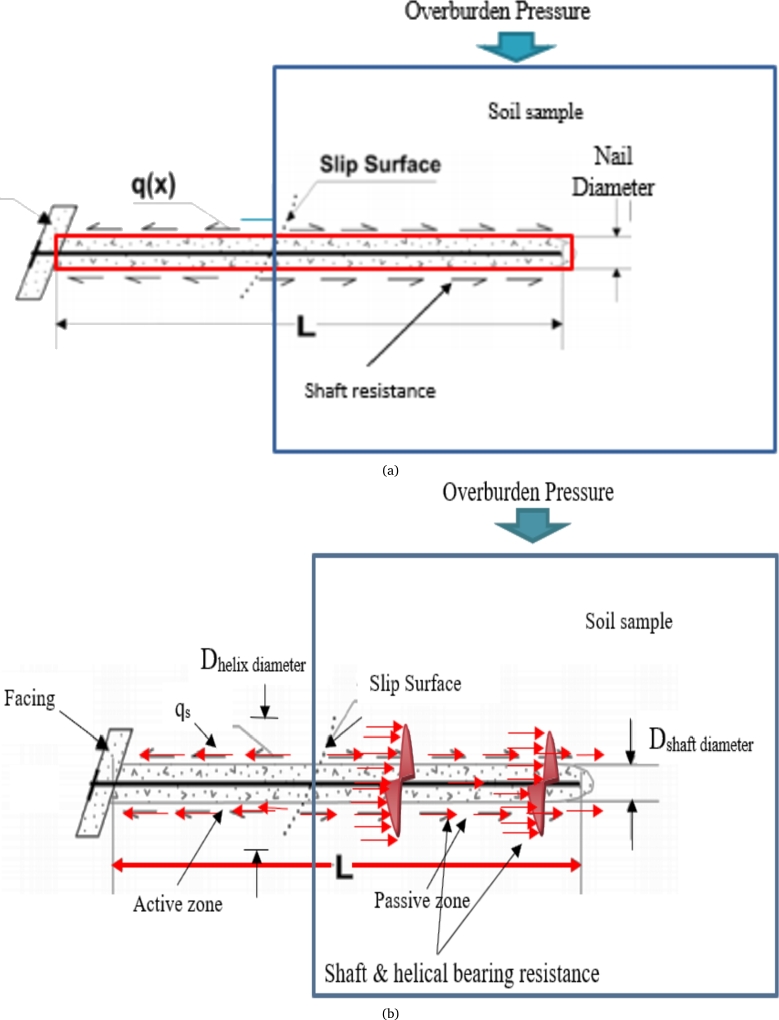
(a) Various forces acting on CN. (b) Various forces acting on HN.

K_a_ is the coefficient of active earth pressure, $K_{a}=\frac{1-sin\emptyset }{1+sin\emptyset }$

w is the weight of soil mass (*γ*h);

q is overburden pressure (i.e., In present study 10 kPa, 20 kPa, and 40 kPa);

Ψ, an inclination of failure plane $=45^{\circ }+\frac{\emptyset }{2}$

i is nail inclination (in present case i=0);

Equation (10) has the limitation that the equation calculates an equal factor of safety for different kinds of nail specimens. In other words, the equation suggested by FHWA, [1] is only suitable for conventional soil nails, the equation cannot forecast accurate the factor of safety for soil nails with a different profile or different geometry. Also, the equation can simulate the installation effect, lateral pressure, and three-dimensional effect of preferable soil, thus the equation is unsuitable for the calculation of the factor of safety for helical soil nails. In the current study, the authors have used the idea of a helical pile or anchor for the calculation of FOS of helical soil nails also abridge the uniqueness of the current study. Fig. 7(a) and Fig. 7(b) show the different forces acting over the conventional and helical soil nail in the present study.

The FOS for a different type of soil nail structure was calculated using the relation given in Eqn. (11).

(11) $$F_{s}=\frac{F_{u}}{{\mathrm{FOS}}_{n}}$$

F_s_ is pullout load (in kN);

$F_{u}$ is the ultimate capacity of the helical soil nail (HN) (in kN);

(FOS)_n_ is the factor of safety;

As in the present analysis, the ratio of helical spacing and helical diameter was adopted as 3, hence the failure will occur as cylindrical shear failure [8], [9], [10], [11], [12]. The ultimate pullout capacity for HN when it is cylindrical shear failure is given as in Eqn. (12).

(12) $$F_{u}=2\pi Rh_{s}\left(c+K_{0}q\tan\Phi \right)+A_{h}\left(cN_{c}+qN_{q}\right)$$

R is the average radius of helices;

$h_{s}$ is helical spacing;

c is cohesion;

Ko is the coefficient of earth pressure at rest = 1 – sin*ϕ*;

q is overburden pressure;

$A_{h}$ is area of bigger helix;

$N_{q}$, $N_{c}$ is Meyerhof's Dimensionless Bearing Capacity Factors calculated by Eqn. (13) and Eqn. (14)

(13) $$N_{q}=1+0.56{\left(12\Phi \right)}^{\Phi /54}$$

(14) $$N_{c}=\left(N_{q}-1\right)\cot\Phi$$

The ultimate pullout capacity for CN is given as in Eqn. (15)

(15) $$F_{u}=\pi d_{s}L_{N}\sigma_{avg}\tan\delta$$

where $\sigma_{avg}$ is average normal stress can be calculated using Eqn. (16) [10], [11], [12], [13], [14], [15], [16]

(16) $$\sigma_{avg}=\frac{\sigma_{v}+\sigma_{h}}{2}=\frac{\sigma_{v}\left(1+K_{0}\right)}{2}=\frac{\sigma_{v}\left(2-\sin\Phi \right)}{2}$$

$\sigma_{v}$, $\sigma_{h}$ are vertical and horizontal stress.

Based on the above analysis the estimated factor of safety for CN was found 1.19, 1.25, and 1.26 under surcharge pressure of 10 kPa, 20 kPa, and 40 kPa respectively. Similarly, FOS for helical soil nails was found 1.23, 1.54, and 1.90 under a similar surcharge pressure (Table 3). As evident From Table 3, under similar surcharge pressure, soil condition, and the same installation depth the HN offers considerably more factors of safety than CN. Also, clear that with an increase in overburden pressure the factor of safety increase slightly for CN, while significantly for HN. As a result, it is clear that helical soil nails function substantially better than conventional soil nails in c-Φ soil.

**Table 3:** Factors of safety for Conventional and Helical Soil Nail.

Overburden pressure (kPa)	10	20	40
Factor of safety for CN	1.19	1.25	1.26
Factor of safety for HN	1.24	1.54	1.70

## 5 Numerical modeling

### 5.1 Model geometry and material properties

PLAXIS 2D, which employs the Finite Element Method (FEM) was utilized to simulate the laboratory model. PLAXIS 2D implemented all of the geometric parameters used in the laboratory-like dimension of the tank, soil nail geometry, and soil characteristics. The Mohr-Coulomb (MC) model was utilized to simulate the laboratory model in PLAXIS 2D software. Both Hooke's and Coulomb's failure theories are included in the MC model. An elasto-plastic model is used to model the soil and nail, which is based on deformation. Table 4 lists the different parameters used in the MC model.

**Table 4:** Soil and Nail parameters adopted in PLAXIS 2D.

Parameters	Values (units)
Modeling element	Plate
Modulus of elasticity of nail (E_n_)	160 GPa (From Exp. load-displacement)
Model type	Elasto-plastic
EA	5.024⁎10^−05^ kN/m
EI	1.7⁎10^−09^ kN- m^2^/ m
Plate diameter	16 mm
Geotechnical properties	Refer to Table 1

The model is based on an undrained plane strain condition. The soil sample's shear strength parameters (c' and *ϕ*') were determined using a consolidated undrained test (CU test). The soil nail element is modeled using the plate elements [8]. The properties of the material were attained on the plate element in such a way that it resembles the axial stiffness and flexural rigidity of a soil nail. The equivalent modulus of elasticity (E_eq_) of soil nail is given as in Eqn. (17):

(17) $$E_{eq}=E_{n}\left(\frac{A_{n}}{A}\right)+E_{g}\left(\frac{A_{g}}{A}\right)$$

E_n_, E_g_ is the modulus of elasticity of nail material and grout respectively,

A_n_ is the cross-sectional area,

A_g_ is a cross-sectional area of CN,

A is the gross area of soil nail.

For grout-free soil nails the component A_g_ and E_g_ are equal to zero, thus Eqn. (21) changes accordingly.

The axial stiffness (EA) and bending stiffness (EI) are given as in Eqn. (18) & (19), over which the FEM works.

(18) $$EA=\frac{E_{n}}{S_{h}}\left(\frac{\pi D_{n}^{2}}{4}\right)$$

$D_{n}$ is overall nail diameter,

$S_{h}$ is unit spacing,

(19) $$\mathrm{EI}=\frac{E_{n}\left(\pi d_{n}^{4}\right)}{S_{h}64}$$

d_n_ is shaft diameter.

The boundary conditions are modeled as standard fixities, where the base of the soil mass is fixed in the x-y direction using Plaxis 2D, but the face is independent of both directions. Interaction between soil and nail is determined by the Strength Reduction Factor (R_inter_). The virtual thickness factor (Δ), which is utilized to construct the mesh, is set to 0.1 to guarantee adequate soil-nail interaction.

The R_inter_ refers to interface friction between soil-nail for sandy soil and fine-grained soil as given in Eqn. (20) and Eqn. (21) respectively:

(20) $$R_{inter}=\frac{\tan\phi_{interface}}{\tan\phi_{soil}}$$

(21) $$R_{inter}=\frac{c_{interface}}{c_{soil}}$$

The analysis was run over the PLAXIS 2D using medium-mesh around the HN and CN. The initial stresses were then accomplished using the K_0_- procedure, which simulates the rest condition of earth pressure. Thus, the model is then examined for safety and deformation for both CN and HN. The Plaxis 2D estimates the factor of safety using the strength-reduction technique resulting in the shear strength of the interface being reduced until the critical or failure value [8]. The strength of CN and HN elements is independent of the phi/c reduction method during the analysis. The algorithm defines the safety factor by the mean of the total multiplier (∑ Msf) define as in Eqn. (22).

(22) $$\sum Msf=\frac{\tan\emptyset_{input}}{\tan\emptyset_{reduced}}=\frac{c_{input}}{c_{reduced}}$$

### 5.2 Factor of safety (FOS) by FEM

**Figure 8:**
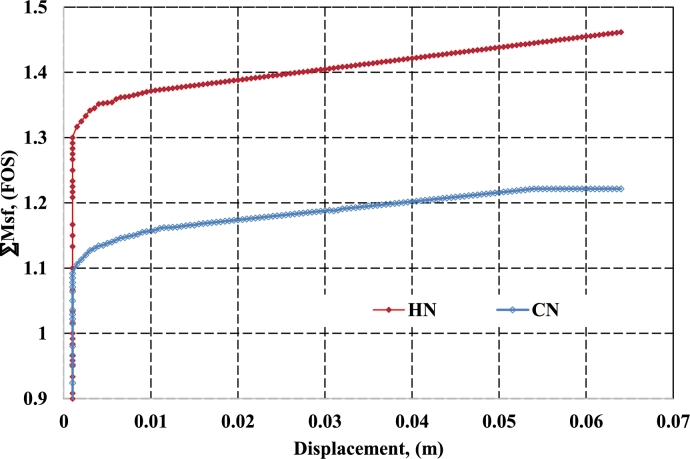
Factor of Safety for CN and HN.

**Figure 9:**
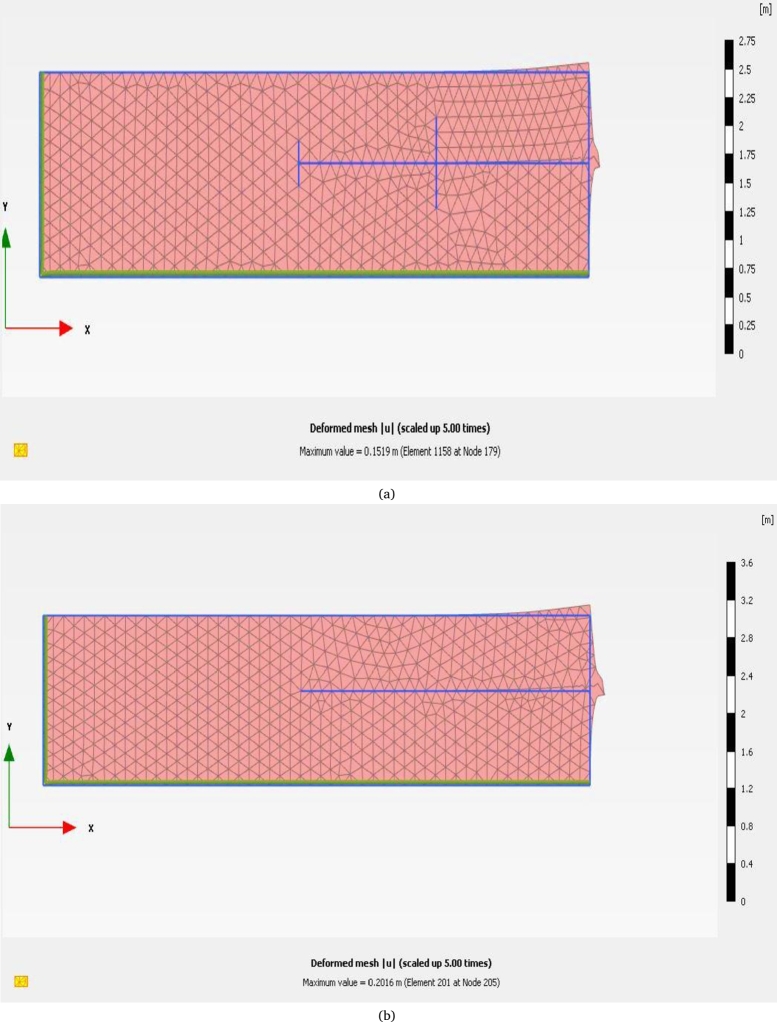
(a). Maximum deformation for HN. (b). Maximum deformation for CN.

Based on the recommendations of the Plaxis guide [21], [22], [23], the FOS is characterized by ∑Msf with displacement factor (|U|m) of soil mass. Nevertheless, the displacement factor is independent of FOS it just revealed whether the critical stage of failure has been established or not. As evident from Fig. 8, the factor of safety of HN and CN was found 1.47 and 1.23 respectively under similar loading conditions. As it is clear that helical soil nail (HN) offers about 19% more safety or FOS than conventional soil nail under similar loading and soil conditions. A similar type of observation was also reported was observed by theoretical analysis. In addition, from Fig. 9(a) and Fig. 9(b) it is observed that under similar conditions the maximum deformation for HN and CN was recorded at 0.15 m and 0.20 m respectively. From the results it is clear that HN offers 5% lesser deformation to the soil mass in comparison to CN, this may be due to the extra bearing resistance offered by the helices of HN. Thus, based on both theoretical and numerical analysis it is clear that helical soil nail offers a predominant factor of safety over conventional soil nails.

## 6 Limitations

The water content of the soil in this study has not remained constant, and also maintaining the water content during the entire study is not practically possible. Thus, the testing has been done on c-Φ soil by measuring the initial water content. Furthermore, mathematical models are not able to incorporate the effect of water content on the soil nail's pull-out load. The boundary conditions are modeled in this study using conventional fixities from the PLAXIS 2D software. Base of the soil slope is fixed in the x-y direction, but back of the soil slope is fixed only in the x-direction and free to move in the y-direction. Face of the soil slope may move freely in both the x and y directions. Top of the soil slope is likewise free to move up and down in the vertical direction. All the boundary conditions were available for soil mass, whereas there was no such provision for soil nail element. Thus, during the analysis, the soil mass and soil nails were considered as a single unit which will predict the result on a little higher side.

## 7 Conclusions

• From both experimental and mathematical study evident that, the pullout shear stress varies linearly with surcharge pressure, which shows that pullout force follows the Mohr-Coulomb trend for c-Φ soil.
• Based on average theoretical and experimental results, two different equations were developed for predicting the pullout force/shear stress for the HN and CN under different surcharge pressures. Experimental and theoretical results indicate that empirical models can predict the peak resistance of helical nails.
• At the time of installing a helical soil nail (HN), soil stresses are progressive while a conventional soil nail (CN) causes significant soil stress drops. Thus, it is clear that conventional soil nails exhibit more disturbance than helical soil nails.
• Helical soil nail (HN) offers about 19% more safety than conventional soil nail (CN) under similar loading and soil conditions. In addition, HN offers 5% lesser deformation to the soil mass in comparison to CN.

## Declarations

### Author contribution statement

Archita Goyal: Conceived and designed the experiments; Performed the experiments; Analyzed and interpreted the data; Contributed reagents, materials, analysis tools or data; Wrote the paper.

Amit Kumar Shrivastava: Analyzed and interpreted the data; Contributed reagents, materials, analysis tools or data; Wrote the paper.

### Funding statement

This research did not receive any specific grant from funding agencies in the public, commercial, or not-for-profit sectors.

### Data availability statement

Data will be made available on request.

### Declaration of interests statement

The authors declare no conflict of interest.

### Additional information

No additional information is available for this paper.
